# Prebiotic feeding elevates central brain derived neurotrophic factor, N-methyl-d-aspartate receptor subunits and d-serine^☆^

**DOI:** 10.1016/j.neuint.2013.10.006

**Published:** 2013-12

**Authors:** Helene M. Savignac, Giulia Corona, Henrietta Mills, Li Chen, Jeremy P.E. Spencer, George Tzortzis, Philip W.J. Burnet

**Affiliations:** a Department of Psychiatry, University of Oxford, Warneford Hospital, Oxford OX3 7JX, UK; b School of Chemistry, Food and Pharmacy, University of Reading, Reading RG2 9AR, UK; c Clasado Research Services Ltd., Reading RG6 6BZ, UK

**Keywords:** *Bifidobacteria*, Dentate gyrus, Glutamate, HPLC, Western blot, Amino acids

## Abstract

The influence of the gut microbiota on brain chemistry has been convincingly demonstrated in rodents. In the absence of gut bacteria, the central expression of brain derived neurotropic factor, (BDNF), and N-methyl-d-aspartate receptor (NMDAR) subunits are reduced, whereas, oral probiotics increase brain BDNF, and impart significant anxiolytic effects. We tested whether prebiotic compounds, which increase intrinsic enteric microbiota, also affected brain BDNF and NMDARs. In addition, we examined whether plasma from prebiotic treated rats released BDNF from human SH-SY5Y neuroblastoma cells, to provide an initial indication of mechanism of action.

Rats were gavaged with fructo-oligosaccharides (FOS), galacto-oligosaccharides (GOS) or water for five weeks, prior to measurements of brain BDNF, NMDAR subunits and amino acids associated with glutamate neurotransmission (glutamate, glutamine, and serine and alanine enantiomers). Prebiotics increased hippocampal BDNF and NR1 subunit expression relative to controls. The intake of GOS also increased hippocampal NR2A subunits, and frontal cortex NR1 and d-serine. Prebiotics did not alter glutamate, glutamine, l-serine, l-alanine or d-alanine concentrations in the brain, though GOSfeeding raised plasma d-alanine. Elevated levels of plasma peptide YY (PYY) after GOS intake was observed. Plasma from GOS rats increased the release of BDNF from SH-SY5Y cells, but not in the presence of PYY antisera. The addition of synthetic PYY to SH-SY5Y cell cultures, also elevated BDNF secretion.

We conclude that prebiotic-mediated proliferation of gut microbiota in rats, like probiotics, increases brain BDNF expression, possibly through the involvement of gut hormones. The effect of GOS on components of central NMDAR signalling was greater than FOS, and may reflect the proliferative potency of GOS on microbiota. Our data therefore, provide a sound basis to further investigate the utility of prebiotics in the maintenance of brain health and adjunctive treatment of neuropsychiatric disorders.

## Highlights

• Prebiotic feeding elevated BDNF and NR1subunit mRNAs, in the rat hippocampus.
• The GOS prebiotic increased cortical NR1, d-serine, and hippocampal NR2A subunits.
• GOS feeding elevated plasma levels of the gut peptide PYY.
• GOS plasma increased BDNF release from human SH-SY5Y neuroblastoma cells.
• BDNF secretion from cells by GOS plasma was blocked by PYY antisera.

## 1 Introduction

There is now compelling evidence for a link between the enteric microbiota and brain function. The proliferation of the *Bifidobacteria* and *Lactobacilli* strains in the large intestine, have anxiolytic and mnemonic effects in both rodents (Li et al., 2009; Bravo et al., 2011) and humans (Messaoudi et al., 2011a,b; Rao et al., 2009; Cryan and Dinan, 2012). The intake of these bacteria as live cultures (probiotics) alters the expression of genes integral to neurodevelopment and complex behaviours in rodents. For instance, the oral administration of *Bifidobacteria* to rats elevated hippocampal brain-derived neurotrophic factor (BDNF) (Bercik et al., 2011a; O’Sullivan et al., 2011), which may underlie some antidepressant actions (Kerman, 2012). At present, only several probiotics have been examined, but it seems likely that of the 40,000 species in the gut (Forsythe and Kunze, 2012), there will be others with psychotropic properties. Thus, intuitively, augmenting the growth of intrinsic gut microbiota with prebiotics (nutrients for intestinal bacteria) may afford greater benefits to the brain (Burnet, 2012).

The prebiotics, fructo-oligosaccharide, (FOS) and galacto-oligosaccharides, (GOS) are soluble fibres which are digested by, and result in the proliferation of, the *Lactobacilli* and *Bifidobacteria* in the gut. Increasing the proportion of these bacteria with prebiotics has many beneficial effects on the gut and the immune system (Drakoularakou et al., 2010; van Vlies et al., 2012; Vulevic et al., 2008, 2013), and increase circulating gut peptides such as glucagon-like peptide-1 (GLP-1) and peptide YY (PYY), which benefit metabolism (Delmee et al., 2006; Overduin et al., 2013). However, the central effects of prebiotic administration have not been explored. Interestingly, selective antimicrobials which elevate the levels of intrinsic gut *Lactobacilli*, also increase brain BDNF concentrations in mice (Bercik et al., 2011a). It is possible therefore, that prebiotic-mediated microbiota proliferation has similar effects, and the measurement of brain BDNF in rodents administered with these compounds would provide the necessary proof-of-principle. Additional evidence suggests that gut bacteria may also influence glutamate neurotransmission in the brain.

Mice devoid of gut microbiota from birth have reduced levels of N-methyl-d-aspartate receptors (NMDARs), specifically the NR1 and NR2A subunits, in the hippocampus (Sudo et al., 2004), or NR2B subunits in the amygdala (Neufeld et al., 2011; Kiss et al., 2012). To our knowledge, the effect of increasing gut microbiota on brain NMDARs has not been explored, and such information may have therapeutic relevance (Collingridge et al., 2013). Interestingly, germ-free mice also lack circulating d-alanine, a *bona fide* NMDAR co-agonist which is rich in bacterial cell walls (Konno et al., 1993), and their inoculation with bacteria restored d-alanine concentrations, which were then increased further by an additional administration of a *Bifidobacteria*. It is reasonable to propose, therefore, that an elevation of central d-alanine, and perhaps other amino acids associated with glutamate neurotransmission, would follow prebiotic administration, and thereby present as a strategy to increase brain NMDAR signalling.

The three major aims of this study were to: (1) test if prebiotic administration to rats altered brain levels of BDNF; (2) examine whether central NMDARs and associated amino acids were altered by prebiotics; and (3) provide initial evidence for neuroactive blood-borne molecules that may affect central BDNF levels after prebiotic feeding. We orally administered water, FOS or GOS to rats for five weeks and measured BDNF NR1, NR2A and NR2B subunits in the frontal cortex and hippocampus, and encoding mRNAs in the hippocampus. The concentrations of glutamate, glutamine, and serine and alanine enantiomers in the plasma, cortex and hippocampus were also quantified. Finally, we measured the levels of PYY and GLP-1 in plasma from prebiotic-fed rats, and tested their effect on BDNF release from SH-SY5Y neuroblastoma cells.

## 2 Materials and methods

### 2.1 Animals

All rat experiments were carried out in accordance with UK Home Office guide lines and under approved licences. Adult male Sprague Dawley rats (225–250 g) were obtained from Harlan Laboratory, UK, and maintained under controlled 12-h light/dark cycle (lights on 7:00 am), temperature (21 ± 1 °C) and humidity (55 ± 5%), with *ad libitum* access to drinking water/fluid and food (standard chow pellets). Rats were weighed at the start and end of experiments.

### 2.2 Prebiotic experiments

Rats were administered a daily oral administration (gavage) of either water, FOS (3 g/kg) or GOS (4 g/kg), for 5 weeks (*n* = 8/group). This dosing regimen was based on previous studies (Anthony et al., 2006). Copies of *Bifidobacteria* spp. genes in DNA extracted from faecal pellets were determined with standard QPCR at the end of the study, as previously described (Ketabi et al., 2011). Twenty-four hours after the last gavage, the animals were sacrificed, their brains removed and trunk blood collected in EDTA-coated tubes. Blood was centrifuged (5000 rpm, 15 min) to obtain plasma which was then stored at −80 °C. The frontal cortex and hippocampus were dissected out from half of the harvested brains. Brain hemispheres and isolated regions were snap-frozen in isopentane on dry-ice and stored with plasmas at −80 °C prior to use. Additional faecal pellets were collected from each animal (*n* = 8/group), weighed, homogenised in PBS (1:1, w/v), and then centrifuged at 14,000 rpm for 10 min at 4 °C. Supernatants were removed and stored at −80 °C prior to HPLC analysis.

### 2.3 Glucose and gut hormone measurements

The concentration of blood glucose was measured in all plasma samples using a GlucoMen LX, blood glucose meter (A. Menarini Diagnostics, UK). Commercial ELISA kits were used to measure plasma PYY (Abnova, UK) and GLP-1 (Millipore, UK), and were performed according to manufacturer’s recommendations.

### 2.4 BDNF analysis

Cortex and hippocampus tissue from all groups (*n* = 8 rats/group) were homogenised in RIPA buffer (1:10 w/v, Sigma–Aldrich, UK) containing protease inhibitors (‘Complete-Mini’, Roche). Protein concentrations were determined using the Bradford reagent (Sigma, UK). Samples of protein extracts were diluted 1:5 v/v in assay buffer, prior to their analysis with a commercial BDNF ELISA kit (BDNF Emax immunoassay system, Promega, UK). Samples of cell culture medium were first diluted in an equal volume of RIPA, and then further diluted (1:5) in BDNF assay buffer. The BDNF ELISA was performed according to manufacturer’s recommendations.

### 2.5 Western blotting

Western blots were performed as previously described (Burnet et al., 2011). Briefly, equal concentrations of protein extracts of cortex or hippocampus (20 μg) from prebiotic and control groups (*n* = 8 rats/group) were mixed with loading buffer (50 mM 1,4-dithiothreitol and 0.025% bromophenol blue), and fractionated with a molecular weight marker (GE Healthcare, Buckinghamshire, UK) by electrophoresis on pre-cast 7.5% SDS/polyacrylamide gels (Biorad, UK), and trans-blotted onto polyvinyl difluoride (PVDF) membranes (Immobilon-P, Millipore, Watford, UK).

The membranes were blocked with 5% (w/v) non-fat milk in PBS containing 0.1% Tween^20^ (PBST) for 45 min, and then incubated for 1 h at room temperature in incubation buffer (PBST with 2% [w/v] milk) containing a primary antibody (diluted 1:1000) against one of three NMDAR subunits: NR1 (AB9864, Millipore, UK), NR2A (AB1555, Millipore, UK) and NR2B (AB15362, Millipore, UK), and β-actin (Sigma–Aldrich, UK, diluted 1:50,000). Membranes were then washed three times for ten minutes in PBST and incubated for 30 min in HRP-linked secondary antibody in blocking buffer. Immunoreactive bands were visualized by chemiluminescence using the ECL-Plus kit (GE Healthcare, Buckinghamshire, UK), and apposing membranes to X-ray film (Kodak BioMax AR film). All antibodies produced a single band of expected molecular weight. The optical densities (OD) of bands were measured using the AlphaImager 3400, and the data expressed as OD ratios of NMDAR subunit:β-actin.

### 2.6 In situ hybridization histochemistry (ISHH)

The frozen rat brain hemispheres were coronally sectioned (14 μm) on a cryostat, and every three sections thaw-mounted onto Superfrost-plus slides (Fisher Scientific, UK). All slides were stored at −80 °C prior to use. Slides containing sections of the dorsal hippocampus [−3.2 to −3.8 mm from Bregma, (Paxinos and Watson, 1986)], were pre-treated as described (Eastwood et al., 1995). One slide from each group (‘water’, ‘FOS’ and ‘GOS’, *n* = 8/group) was used for ISHH analysis.

Commercially synthesized (MWG, UK) oligodeoxyribonucleotides complementary to: BDNF (bases 883–927, NM001270630.1), NR1 (bases 746–780, NM008169.1), NR2A (bases 1642–1676, NM008170.2) or NR2B (bases 1758–1792, NM010350.2) were used in an establish ISHH method (Eastwood et al., 1995). Oligodeoxyribonucleotide probes were 3′-end labelled with [^35^S]-dATP using terminal deoxynucleotidyl transferase (Promega, UK). Probes were diluted in hybridization buffer, pipetted onto the tissue sections (1 × 10^6^ cpm/section), cover-slipped and then incubated for >16 h at 34 °C lidded Perspex trays lined with filter paper soaked with 4× SSC/50% formamide.

Post-hybridization washes included: 2× SSC rinse at room temperature to remove cover-slips; 0.5× SSC, 20 min (3×) at 55 °C; 0.5× SSC 30 min (2×) at room temperature. Slides were rinsed in ddH_2_O, dried and apposed to X-ray film (Kodak, Biomax MS) for 7–28 days with ^14^C-microscales. Average grey densities over the dentate gyrus, CA1, and CA3 subfields of the hippocampus in the three sections from each group were measured for each of the mRNAs using computer-assisted image analysis, and were calibrated to ^35^S nCi/g tissue equivalents using the commercial ^14^C-microscales and a ^14^C to ^35^S conversion factor of 3.0 (Eastwood et al., 1995).

### 2.7 HPLC analysis

Small fragments of the cortical and hippocampal tissue (50 mg) were individually homogenised in ice-cold methanol (1:10 w/v) and centrifuged at 14,000 rpm for 10 min at 4 °C. Supernatants from faecal homogenates and plasma samples were mixed with 3 volumes of methanol and also centrifuged for 10 min at 4 °C. Supernatants (10 μl) from tissue or faecal homogenates or plasma were subjected to online, pre-column, derivatization (Grant et al., 2006), by injecting them onto a Hewlett–Packard 1100 liquid chromatography (Agilent Technologies, Palo Alto, CA), with an equal volume of derivatizing reagent [o-phthaldialdehyde (2 mg) and Boc-l-cysteine (2 mg) in 0.2 ml of methanol and 0.8 ml of 0.4 M of sodium borate buffer (pH = 9)], for 5 min prior to column separation. Separation was achieved using an Agilent Zorbax Eclipse XDB-C18 column (4.6 × 150 mm, 5 μm) maintained at 30 °C and a separation protocol similar to that of (Morikawa et al., 2001). The mobile phases consisted of acetonitrile (phase A) and 100 mM sodium acetate buffer pH = 6 (phase B) and were pumped through the column at 1.4 ml/min. The following gradient system was used (min/% B): 0/91, 35/84, 65/84. Detection of derivatized amino acids was by fluorescence detection (emission: 443 nm; excitation 344 nm). Eight point calibration curves of the d- and l-amino acids (Sigma–Aldrich, UK) were constructed using authentic standards (0.5–1000 pmol) and in each case were found to be linear with correlation coefficients of >0.995.

### 2.8 Cell culture

The release of BDNF from human SH-SY5Y neuroblastoma cells was investigated using a recently described protocol (Coco et al., 2013). Briefly, cells were cultured in Dulbecco’s Modified Eagle Medium (DMEM; Sigma, Poole, UK) supplemented with 10% foetal calf serum (Sigma), 2 mM l-glutamine (Sigma) and 1% non-essential amino acids (Sigma), maintained in a humidified incubator at 37 °C and 5% CO_2_. Prior to release experiments, 24-well plates were seeded with 1 × 10^5^ cells/well and incubated for 24 h. Plasma (50 μl) from rats fed water, FOS or GOS for 5 weeks (see above), containing either IgG or anti-PYY antisera (1:200, Abnova, UK) were then added to the 0.5 ml of culture media for 4 h. A total of 24 plasma samples (8 plasmas/group) were tested in triplicate. Some cells were incubated with synthetic PYY peptide (20 nM) alone. This experiment was then repeated in the presence or absence of GLP-1 antisera (Millipore, UK) or GLP-1 peptide. All media was removed following incubations, and stored at −80 °C prior to BDNF assays.

### 2.9 Data analysis

All data were expressed as mean ± standard error of the mean (SEM). Statistical comparisons between groups from rat experiments were performed with one-way ANOVA followed by *post hoc* analysis (Tukey HSD). Cell culture data were analysed non-parametrically (Kruskall–Wallis), followed by *post hoc* Mann–Whitney *U* tests.

## 3 Results

### 3.1 Rat faecal *Bifidobacteria* after prebiotics

The numbers of *Bifidobacteria* in faecal pellets from FOS-fed rats were significantly greater than controls in an ANOVA and *post hoc* (Tukey HSD) analysis i.e. controls: 2.38 × 10^9^ ± 0.23 × 10^9^ *vs* FOS: 2.98 × 10^9^ ± 0.22 × 10^9^, *p* < 0.05), whereas the numbers of *Bifidobacteria* from GOS-fed animals were significantly greater than both controls and FOS-fed rats i.e. controls: 2.38 × 10^9^ ± 0.23 × 10^9^ *vs* GOS: 4.28 × 10^9^ ± 0.43 × 10^9^, *p* < 0.01; and FOS: 2.98 × 10^9^ ± 0.22 × 10^9^ vs GOS: 4.28 × 10^9^ ± 0.43 × 10^9^, *p* < 0.05. Thus, the percentage increase of *Bifidobacteria* after FOS and GOS administration relative to water intake were approximately,+25% and +80%, respectively.

### 3.2 The effect of prebiotics on BDNF and NR1 in the rat frontal cortex and hippocampus

**Fig. 1:**
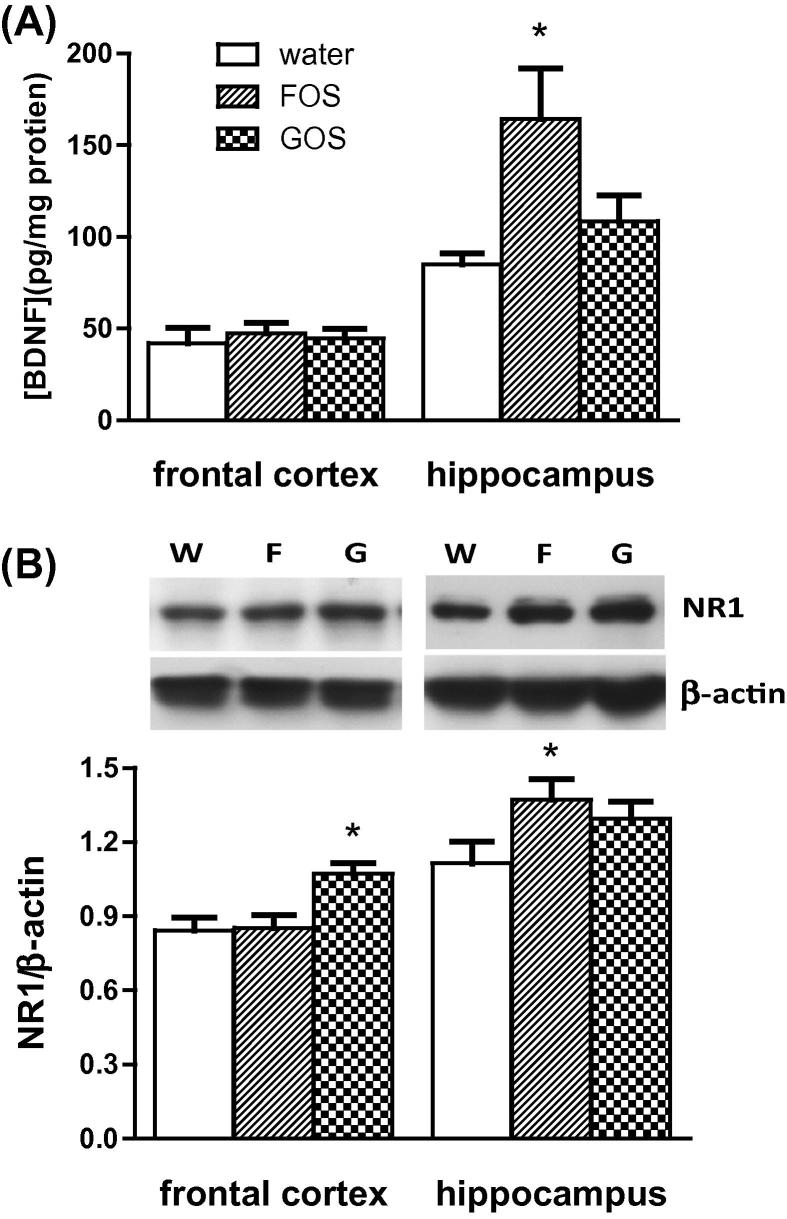
The effect of an oral administration of water or prebiotic (FOS or GOS) on the levels of (A) BDNF and (B) NR1 subunits in the cortex and hippocampus of the rat. Representative Western blot images of NR1 and β-actin immunoreactivity in protein extracts from water (W), FOS (F) and GOS (G)-fed rats are shown in (B, inset). NR1 levels were expressed as a ratio of β-actin. ^∗^*p* < 0.05 (*n* = 8/group).

The levels of BDNF protein in extracts of frontal cortex did not differ between groups (Fig. 1A). However, BDNF in hippocampal extracts of FOS administered rats were significantly higher than those of control and GOS fed animals. Western blots revealed that GOS-fed rats contained significantly greater levels of NR1 immunoreactivity in the frontal cortex compared to control and FOS animals (Fig. 1B). Analysis of the hippocampus, however, revealed that FOS rats contained significantly more NR1 subunits than the other groups, though an increased trend (*p* = 0.058) was observed in GOS animals relative to controls.

### 3.3 The effect of prebiotics on NR2A and NR2B subunits in the rat frontal cortex and hippocampus

**Fig. 2:**
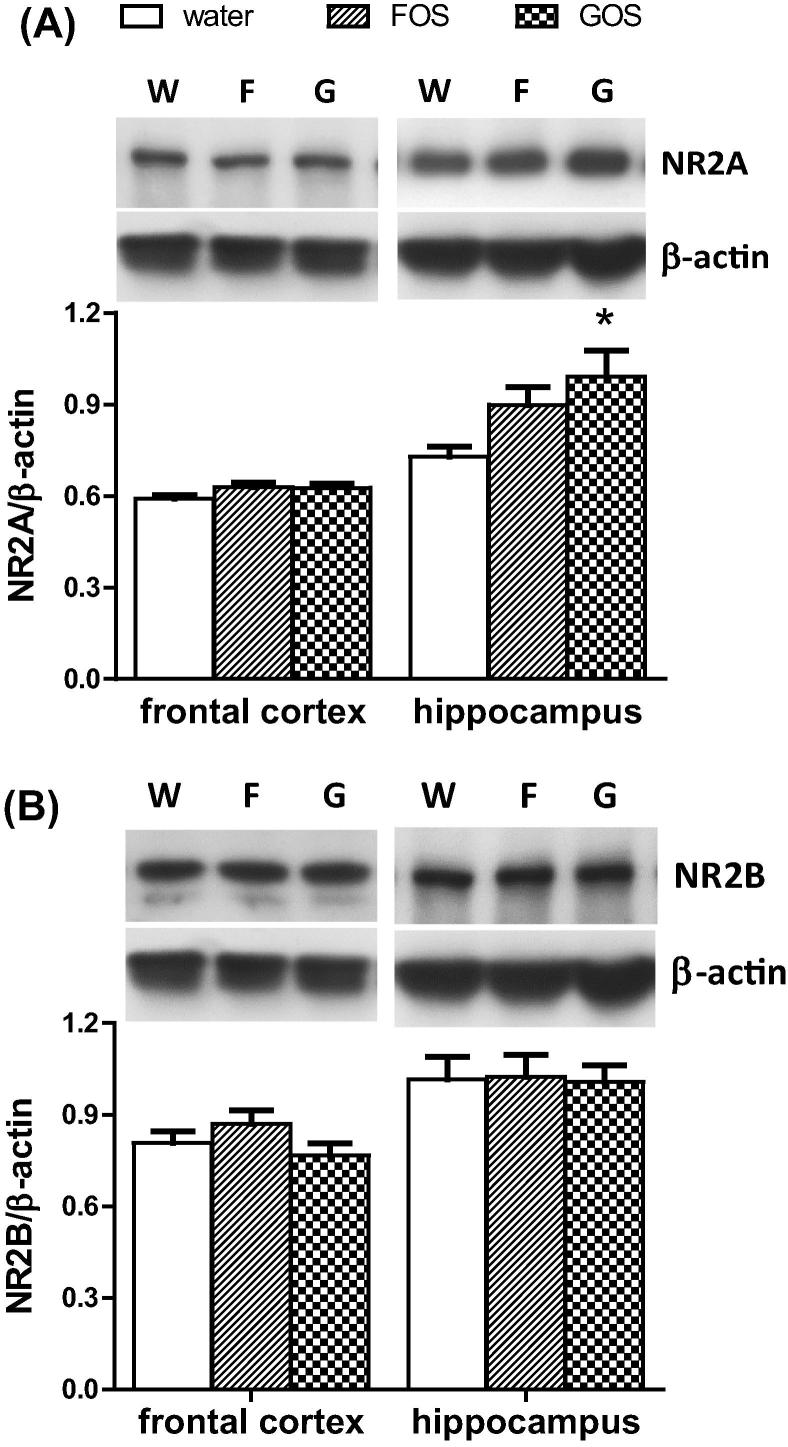
The effect of an oral administration of water or prebiotic (FOS or GOS) on the levels of (A) NR2A and (B) NR2B subunits in the cortex and hippocampus of the rat. Representative Western blot images of NR subunits and β-actin immunoreactivity in protein extracts from water (W), FOS (F) and GOS (G)-fed rats are shown (inset). NR2 subunit levels were expressed as a ratio of β-actin. ^∗^*p* < 0.05 (*n* = 8/group).

On Western blots hippocampal, but not cortical, extracts from GOS-fed animals, contained significantly greater NR2A immunoreactivity compared to controls (Fig. 2). The level of NR2B in the frontal cortex and hippocampus, was not affected by prebiotic feeding.

### 3.4 The effect of prebiotics on BDNF and NR subunit mRNAs in the hippocampus

**Fig. 3:**
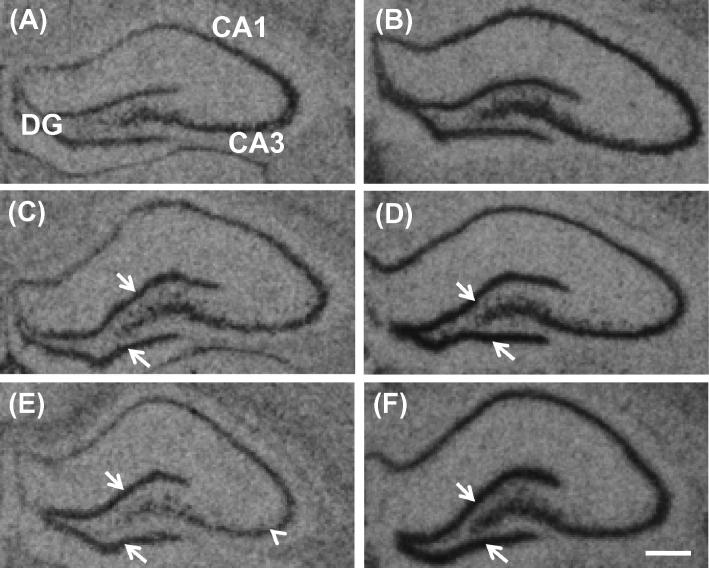
Representative autoradiographs of BDNF (A, C and E) and NR1 subunit (B, D and F) mRNA expression in the rat hippocampus following an oral administration of water (A and B), FOS (C and D) or GOS (E and F). Arrows delineate increased expression, and arrow head indicates reduced expression. DG = dentate gyrus, CA1 and CA3 = Cornu Ammons subfields of the hippocampus. Scale bar = 200 μM.

**Fig. 4:**
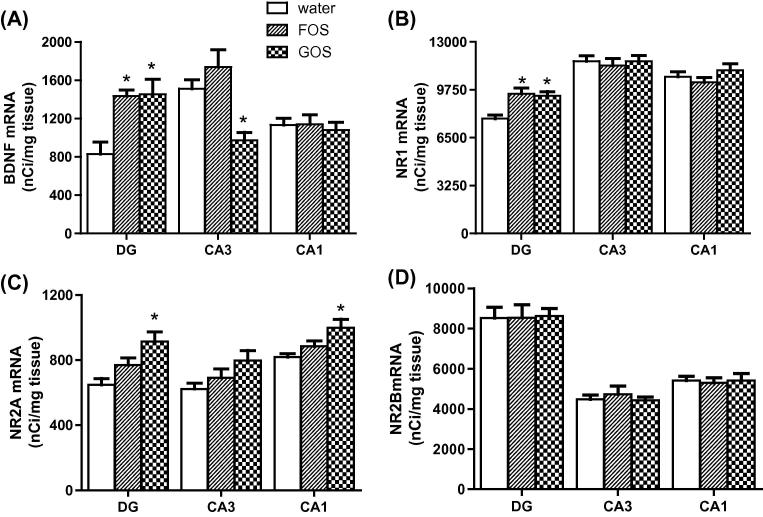
Prebiotics (FOS, GOS) differentially alter the abundance of mRNAs encoding BDNF (A), NR1 (B), NR2A (C), but not NR2B (D) subunits. DG = dentate gyrus, CA1 and CA3 = Cornu Ammons subfields of the hippocampus. ^∗^*p* < 0.05 (*n* = 8/group).

Prebiotic administration increased the abundance of BDNF (Figs. 3A, C, E and 4) and NR1 (Fig. 3B, D and F) mRNAs in the dentate gyrus of the hippocampus, relative to controls. A reduction of BDNF mRNA in the CA3 subfield of GOS-fed rats was also observed (Fig. 3C). Densitometry confirmed significantly greater BDNF and NR1 expression in the dentate gyrus of prebiotic rats (Fig. 4A and B). The administration of GOS resulted in an elevation of NR2A (Fig. 4C), but not NR2B (Fig. 4D), mRNA in the dentate gyrus and CA1 subfield relative to controls and FOS-fed animals.

### 3.5 Faecal, plasma and brain amino acid concentrations after prebiotics

This study tested whether an elevation of gut bacteria increased central d-alanine concentrations by elevating the amounts of this d-amino acid in the gut and the circulation. The concentrations of free d-alanine in faecal pellets of GOS fed rats were significantly greater than control and FOS animals, with FOS administration resulting in intermediate levels of this d-amino acid (Table 1). Plasma d-alanine levels were significantly higher in GOS-fed rats compared to control animals (Table 1), and a slight, though not significant (*p* = 0.086), increase was observed in FOS-fed rats. Prebiotic administration did not alter the concentrations of other circulating amino acids (Table 2). Rats fed with GOS had a significantly higher concentration of d-serine in the frontal cortex compared to controls (Table 2), though the levels of all other amino acids remained unchanged after prebiotic feeding.

### 3.6 Body weight change, and plasma glucose, BDNF, PYY and GLP-1after prebiotics

The administration of GOS significantly increased the concentration of plasma PYY, but neither FOS nor GOS significantly altered circulating GLP-1 (Table 3). An increased trend (*p* = 0.077) was noted in the plasma of FOS-fed rats. Glucose levels, body weight and plasma BDNF were not significantly changed after prebiotic feeding.

### 3.7 Correlations

There was a significant correlation between the number of faecal *Bifidobacteria* and frontal cortex NR1 protein (Pearson’s *r* = 0.713, *p* < 0.05), in a pooled analysis of control and experimental animals (*n* = 24). There was also an overall correlation between microbiota numbers and plasma d-alanine (*r* = 0.54, *p* < 0.05) and l-alanine (*r* = 0.52, *p* < 0.05). No other parameter measured in either plasma or brain correlated with faecal *Bifidobacteria* numbers. There was an overall significant correlation between the levels of cortical d-serine and NR1 protein (Pearson’s *r* = 0.684, *p* = 0.01). Individual group analysis revealed that this association was only significant after GOS feeding (GOS: *r* = 0.96, *p* = 0.04; FOS: *r* = 0.68, *p* = 0.32; water: *r* = 0.01, *p* = 0.989).

### 3.8 The effect of plasma from prebiotic-fed rats on BDNF levels in SH-SY5Y cells

**Fig. 5:**
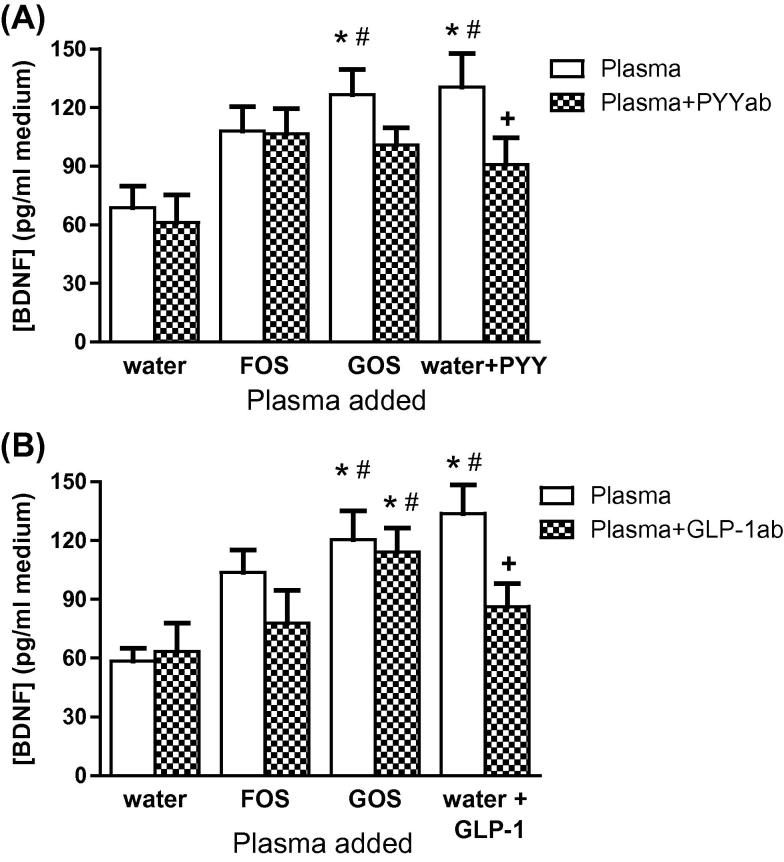
The effect of prebiotic-fed rat plasmas on BDNF release from SH-SY5Y cells, in the absence or presence of PYY (A) or GLP-1 (B) antisera. ^∗^*p* < 0.05, compared to cells exposed to plasma from water-fed rats; ^#^*p* < 0.001, compared to cells exposed to plasma from water-fed rats containing PYY or GLP-1 antisera; ^+^*p* < 0.05, compared to cells exposed to plasma from water-fed rats containing PYY or GLP-1 peptide (*n* = 8 plasmas/group).

The addition of plasma from rats fed with GOS, elevated the extracellular concentrations of BDNF compared to controls (Fig. 5A). These levels were similar to those from cells exposed to synthetic PYY. A non-significant increase of BDNF release was also noted after the addition of FOS plasma. In the presence of PYY antisera BDNF secretion by GOS plasma did not reach significance. In another experiment, the presence of GLP-1 antisera did not affect BDNF secretion from cells by GOS plasma (Fig. 5B), though a non-significant reduction of BDNF release after the addition of FOS plasma was noted.

## 4 Discussion

Studies have shown that probiotics have psychotropic effects, but the neurobiological consequences of prebiotic intake have not been explored. The aim of the current study was to provide unequivocal evidence for neurochemical and molecular changes in the rat brain following prebiotic consumption, to prelude future functional analyses. The specific hypotheses tested were, first, that *Bifidobacteria* proliferation by prebiotics is associated with an increase in brain BDNF, as evinced with probiotics; and second, that prebiotic augmentation of commensal microbiota elevates central NMDAR subunits, given that these receptors are reduced in germ-free rodents. Our data support both suppositions and present substantial grounds for exploring the utility of prebiotics in the modulation of brain function. We have also offered an initial indication for the involvement of PYY, and potentially other gut hormones, in the augmentation of BDNF signalling following prebiotic intake. The interpretations and relevance of our findings are discussed in turn.

The elevated expression of BDNF and encoded protein in rats fed with FOS, is consistent with the effect of a *Bifidobacterium* probiotic (Bercik et al., 2011a; O’Sullivan et al., 2011) and the selective proliferation of these species with antimicrobials (Bercik et al., 2011a). Thus, FOS administration may have augmented the colonization of similar psychotropic strains, within the moderate overall increase in *Bifidobacteria* numbers relative to GOS fed rats. In view of these observations therefore, it was surprising that GOS did not alter the levels of hippocampal BDNF protein and, moreover, by a greater magnitude than FOS.

Unaltered BDNF protein after GOS feeding may have reflected the reciprocal change in BDNF mRNA in the dentate gyrus and CA3 region of the hippocampus. If these alterations were translated to protein, then it is unlikely that an overall change in BDNF would be detected in whole hippocampal homogenates. The measure of BDNF in regionally dissected hippocampus would test this possibility. Arguing against a causal link between gut bacterial densities and BDNF gene expression, is a similar increase of BDNF mRNA in the dentate gyrus of both FOS and GOS rats, in spite of the fewer numbers of *Bifidobacteria* in the FOS group. The possibility that prebiotics alter brain signalling independently of the gut microbiota cannot be ruled out, and a direct interaction between oligosaccharides and the gut mucosa has been shown to influence the response of the immune system (Bode et al., 2004; Eiwegger et al., 2010), which may then impact on brain chemistry. The elevation of gut hormones after prebiotic intake might also reflect a direct effect of oligosaccharides on the gut (see below).

The physiological relevance of a reciprocal change in BDNF mRNA in two regions of the hippocampus after GOS intake is difficult to interpret without functional measures. However, an elevation of BDNF gene expression in the dentate gyrus has been associated with antidepressant action (Kerman, 2012). A similar elevation of BDNF mRNA after FOS and GOS administration is, therefore, in keeping with a potential antidepressant/anxiolytic property of gut bacteria (Bercik et al., 2011a). The concomitant decrease of BDNF mRNA in the CA3 is more difficult to interpret, though one possibility is that we were observing the differential, activity-dependent expression of BDNF mRNA splice variants in each hippocampal subfield (Chiaruttini et al., 2008). Regional molecular and electrophysiological analyses of the rat hippocampus after GOS administration are, therefore, required.

The potential mechanisms underlying the elevation of cortical NR1 subunits after GOS feeding remains elusive, and although they correlate with *Bifidobacteria* numbers (see Section 3), elevated NR1 may have also been mediated by the direct physiological response of the gut to GOS such as the release of PYY (see discussion below). Nevertheless, the significant elevation of hippocampal NR1 mRNA after FOS and GOS administration is intuitive in view of data showing reduced NR1 in mice devoid of gut bacteria (Neufeld et al., 2011), though it is not clear why a parallel change in hippocampal NR1 protein occurred only after FOS feeding. Given the potential complexities of prebiotic actions already encountered (cf: reciprocal changes of BDNF mRNA abundance in GOS-fed rat hippocampus), unaltered hippocampal NR1 protein in GOS rats may be authentic, and mechanistically associated with the increase of NR2A subunits, which was not apparent after FOS intake. Altered NR1:NR2 subunit ratios in the rodent hippocampus following pharmacological and genetic manipulations are not unusual (Owczarek et al., 2011; Kato et al., 2012), and in the latter study, an increase of NR2A, but not NR1, subunits is associated with significant functional outcomes. Similarly, unaltered NR2B subunits after prebiotic administration may suggest that *Bifidobacteria* and/or the metabolic response to GOS ingestion may not affect the neurophysiological processes, such as long-term depression, which are associated with dynamic changes in NR2B subunits (Liu et al., 2004). Of course, the lack of functional information to support our interpretations of these data is the major caveat of this study.

The demonstration of elevated faecal and plasma d-alanine concentrations after GOS feeding is consistent with studies showing that gut bacteria, including *Bifidobacteria*, are a source of this d-amino acid (Konno et al., 1993). The significant reduction of blood l/d-alanine ratios confirms that this d-alanine was not directly derived from circulating l-alanine. In spite of this, central d-alanine concentrations remained unaltered after prebiotic feeding, and this may be because, higher plasma concentrations are required to initiate the appropriate kinetics for d-alanine uptake into the brain (Morikawa et al., 2007).

The current investigation also revealed that GOS-fed rats contained greater amounts of cortical d-serine than controls. Since the same animals also showed greater levels of cortical NR1 subunits which are the d-serine binding moiety, it is reasonable to propose that NMDAR mediated signalling was elevated in the frontal cortex. Of course, only direct electrophysiological analysis of NMDAR signalling after GOS feeding can prove this. Of note, the increase of cortical d-serine did not appear to arise from the plasma but may have been synthesized locally, as suggested by a slight, though not significant, rise of cortical l-serine after GOS feeding. That is, the synthesis of d-serine from l-serine by serine racemase (Sikka et al., 2010), may have been a homeostatic response (preservation of l/d-serine ratios) to either local elevations of the l-enantiomer, and/or a greater demand for d-serine. The possibility that d-alanine is linked to an elevation of central d-serine signalling, is suggested by studies demonstrating augmented d-serine release from neurons in the presence of d-alanine (Rosenberg et al., 2010).

We have confirmed that the administration of GOS to rats increases circulating concentrations of PYY (Overduin et al., 2013). It is reasonable to suggest, therefore, that the effects of GOS on brain signalling may be mediated by gut hormones. Earlier work has shown that PYY has the potential to affect the brain either directly (Connor et al., 1997; Nonaka et al., 2003) or via vagal nerve activity (Hernandez et al., 1994). Our *in vitro* data suggest that elevated brain BDNF expression after GOS intake, may have been mediated by plasma PYY. Another study has shown that plasma from mice administered with *Bifidobacterium longum*, did not change the expression of BDNF mRNA in cultured SH-SY5Y cells (Bercik et al., 2011b), and this was reasoned to be further evidence of a vagal, rather than a ‘blood-borne’ mediation of probiotic central effects. It is reasonable to assume that our discrepant data reflect the different parameters measured in both studies (i.e. BDNF release versus gene expression) and/or different actions of *B. longum* and prebiotics on brain BDNF. However, the presence of neuroactive substances in blood does not preclude the involvement of gut-brain vagal circuitry after prebiotic ingestion. The enteric secretion of PYY after GOS intake may directly affect local BDNF signalling in myenteric neurons of the gut (Boesmans et al., 2008), which innately influence vagal nerve activity (Murphy and Fox, 2010). Of course, since circulating PYY can also directly access brain PYY receptors (Hernandez et al., 1994; Nonaka et al., 2003), in all likelihood, the central effects of PYY would involve direct and indirect mechanisms. However, the mechanisms underlying the increase of hippocampal BDNF after FOS intake remain elusive. Whether PYY or, indeed, other factors such as short-chain fatty acids (Jakobsdottir et al., 2013), and/or the immune system (Vulevic et al., 2008, 2013), underlie the changes in BDNF and NMDAR signalling after prebiotics, requires further examination.

## 5 Conclusion

Our results have provided the necessary ‘proof-of-principle’ for the central actions of prebiotic consumption. The increase of hippocampal BDNF after prebiotic intake is consistent with a probiotic effect, and may have been a direct consequence of elevated gut *Bifidobacteria* numbers. However, an additional effect of gut hormones (e.g. PYY) or other mediators, such as the immune system resulting from direct oligosaccharide-gut interactions, cannot be ruled out. The elevation of NMDAR subunits after prebiotics is intuitive given their reduction in the brains of germ-free animals. Furthermore, the strong correlation between *Bifidobacteria* numbers and cortical NR1 levels presented in this report, further supports a link between the microbiota and central glutamate neurotransmission. Mechanistic investigations beyond the scope of the present study, are now required to ascertain the systems underlying the observed changes, and will also reveal if vagal nerve modulation is involved. Moreover, behavioural analysis in rats will ascertain if the changes in BDNF after prebiotics impart an anxiolytic action, or that increased NMDAR subunits translate to improved cognitive performance. Importantly, our study has provided sufficient cause to warrant further exploration into the utility of prebiotics in therapies of neuropsychiatric illness and which, by virtue of their ability to proliferate gut bacteria and stimulate neuroendocrine (and other) responses, may even prove to be more potent than probiotics.

## Conflict of interest

Clasado Ltd, UK made a financial contribution towards the study, as part of a BBSRC scheme.

## Authorship credit

H.M.S., J.P.E.S. and P.W.J.B. made a substantial contribution to the conception and design of the study, and the analysis and interpretation of the data together with G.C., L.C., and H.M. H.M.S. and P.W.J.B. drafted the article, which was critically reviewed for intellectual content by G.T.

## Figures and Tables

**Table 1:** Amino acid concentrations in rat faecal pellets and plasma following a five week oral administration of water or prebiotic.

[Amino acid] (nmol/g faeces) & (nmol/ml plasma)			
Amino acid	Water	FOS	GOS
Faecal pellets			
l-Alanine	7.8 ± 0.3	12.5 ± 0.8⁎	18.7 ± 1.8⁎^,^⁎⁎
d-Alanine	4.2 ± 0.1	5.9 ± 0.2	9.0 ± 0.9⁎^,^⁎⁎
l/d-Alanine	1.9 ± 0.1	2.1 ± 0.1	2.1 ± 0.1
Glutamate	13.1 ± 1.2	20.9 ± 1.2	32.4 ± 3.4⁎^,^⁎⁎
Glutamine	1.6 ± 0.1	2.8 ± 0.2^a^	4.2 ± 0.4⁎^,^⁎⁎
Glut/Gln	8.0 ± 0.2	7.4 ± 0.2	7.8 ± 0.1
l-Serine	3.9 ± 0.2	7.0 ± 0.8	10.7 ± 1.1⁎^,^⁎⁎
d-Serine	0.1 ± 0.01	0.2 ± 0.01⁎	0.2 ± 0.02⁎
l/d-Serine	39.0 ± 1.1	35.0 ± 2.5	53.5 ± 2.2⁎

Plasma			
l-Alanine	299.9 ± 22.1	308.6 ± 21.8	310.0 ± 21.7
d-Alanine	4.0 ± 0.5	5.2 ± 0.4	6.1 ± 0.5⁎
l/d-Alanine	79.2 ± 8.8	61.7 ± 6.9	49.3 ± 1.7⁎
Glutamate	82.6 ± 6.6	78.9 ± 7.3	68.7 ± 1.6
Glutamine	419.0 ± 14.1	420.9 ± 18.7	411.2 ± 17.9
Glut/Gln	0.2 ± 0.02	0.2 ± 0.01	0.2 ± 0.01
l-Serine	141.8 ± 7.8	142.9 ± 5.3	144.0 ± 6.8
d-Serine	2.0 ± 0.1	1.9 ± 0.1	2.0 ± 0.1
l/d-Serine	72.2 ± 2.4	74.5 ± 1.1	72.5 ± 1.6

⁎ *p* < 0.05 compared to water.

⁎⁎ *p* < 0.05 compared to FOS.

**Table 2:** Amino acid concentrations in the rat cortex and hippocampus following a five week oral administration of water or prebiotic.

[Amino acid] (pmol/mg tissue)			
Amino acid	Water	FOS	GOS
Frontal cortex			
l-Alanine	90.5 ± 7.9	100.9 ± 8.4	108.6 ± 6.6
d-Alanine	2.6 ± 0.4	2.4 ± 0.2	2.3 ± 0.3
l/d-Alanine	37.7 ± 5.9	42.8 ± 3.2	42.2 ± 2.0
Glutamate	783.9 ± 75.6	837.3 ± 48.0	822.3 ± 47.9
Glutamine	618.9 ± 52.7	645.4 ± 44.7	700.5 ± 44.0
Glut/Gln	1.27 ± 0.06	1.32 ± 0.1	1.18 ± 0.03
l-Serine	141.4 ± 13.6	152.8 ± 6.0	161.8 ± 10.9
d-Serine	40.3 ± 2.9	48.7 ± 1.7	53.6 ± 3.4⁎
l/d-Serine	3.5 ± 0.23	3.1 ± 0.07	3.0 ± 0.14

Hippocampus			
l-Alanine	99.7 ± 9.5	92.0 ± 6.2	97.5 ± 9.3
d-Alanine	2.8 ± 0.4	2.6 ± 0.2	2.3 ± 0.2
l/d-Alanine	36.9 ± 3.9	36.5 ± 3.7	42.0 ± 2.5
Glutamate	705 ± 79.8	649 ± 59.3	643.9 ± 68.1
Glutamine	545.1 ± 58.2	480.1 ± 52.6	504.6 ± 48.0
Glut/Gln	1.30 ± 0.04	1.38 ± 0.09	1.27 ± 0.05
l-Serine	121.4 ± 12.4	111.4 ± 7.8	111.9 ± 9.3
d-Serine	37.4 ± 4.1	34.6 ± 2.1	35.3 ± 3.4
l/d-Serine	3.3 ± 0.03	3.2 ± 0.04	3.2 ± 0.08

⁎ *p* < 0.05 compared to water.

**Table 3:** Rat body weight change (Δ), plasma glucose, BDNF, GLP-1 and PYY levels after a 5 week oral administration of water, FOS or GOS.

Group			
Parameter	Water	FOS	GOS
DBody weight (g)	85.7 ± 4.3	86.6 ± 4.5	79.0 ± 5.0

Plasma			
BDNF (ng/L)	32.4 ± 6.5	29.0 ± 5.2	30.4 ± 6.2
Glucose (mM)	7.3 ± 0.4	6.9 ± 0.4	7.7 ± 0.3
GLP-1 (ng/L)	201.7 ± 24.1	258.1 ± 26.2	199.4 ± 27.9
PYY (ng/L)	90.7 ± 8.4	94.9 ± 11.8	134.4 ± 12.0⁎

⁎ *p* < 0.05 compared to water.
